# A Randomized Controlled Trial Comparing Subcutaneous Preservation of Bone Flaps with Cryogenic Preservation of Bone Flaps for Cranioplasty in Cases of Traumatic Brain Injury

**DOI:** 10.3390/brainsci15050514

**Published:** 2025-05-17

**Authors:** Rachith Sridhar, Anil Kumar, Harendra Kumar, Abdul Vakil Khan, Abdul Hakeem, Deepak Kumar, Anurag Kumar, Majid Anwer

**Affiliations:** Department of Trauma Surgery and Critical Care, All India Institute of Medical Sciences, Patna 801507, Bihar, India; rachith10862@aiimspatna.org (R.S.); harendra15989@gmail.com (H.K.); khanvakil30@yahoo.com (A.V.K.); drabdhakeem@gmail.com (A.H.); deepaky447@gmail.com (D.K.); anurages@aiimspatna.org (A.K.); majidanwer1987@gmail.com (M.A.)

**Keywords:** brain injuries, traumatic, decompressive craniectomy, cryopreservation, subcutaneous fat, abdominal, surgical wound infection

## Abstract

Background and objectives: Decompressive craniectomy (DC) is a surgical procedure, useful for relieving the intracranial pressure following trauma. Following reduction in cerebral oedema, the bone is placed back to cover the defect. During the interim period, the bone flap may be preserved using cryopreservation or in subcutaneous tissue. This leads to a need to determine the benefits and risks involved in preservation of bone flap in a subcutaneous pocket or conventional freezer following decompressive craniectomy in traumatic brain injury. Materials and methods: An open randomized controlled trial was conducted at a level one trauma centre from July 2023 to December 2024. Simple randomization was performed in order to allocate patients into the subcutaneous preservation group and the cryogenic preservation group. Patients underwent cranioplasty after 3 months and were followed up post-operatively for complications and Glasgow Outcome Scale assessment. Results: The study initially recruited a total of 158 patients, out of which 104 patients remained eligible for the final analysis. The patients with cryopreserved flaps were found to have a higher rate of surgical site infection (31.3%) as compared to those with subcutaneously preserved flaps (5.6%), with the differences being statistically significant (*p* < 0.001). Among the 87 patients who had a poorer Glasgow Outcome Scale (GOS) score before the intervention, 55 (63.2%) patients had at least some improvement in GOS over a period of one month. Conclusion: The use of subcutaneous preservation of bone is more beneficial in resource-limited settings as compared to conventional freezer storage.

## 1. Introduction

Traumatic brain injury (TBI) is a major public health problem. Road traffic injuries, falls from height, and interpersonal violence are the most common causes of TBI. In countries like India, more than 1.5 to 2 million people are injured, and at least 500,000 lives are lost annually. It has a high mortality rate (30%) and is associated with significant morbidity. Lack of awareness, poor implementation of traffic rules like wearing helmets, and falls of high-risk populations like children and the elderly from unfenced roofs and poor road infrastructure have all contributed to the burden [1].

Raised intracranial pressure (ICP) is the most common cause of death in cases of traumatic brain injury (TBI). Multiple medical and surgical methods exist for management of ICP. Increased cerebral edema is a dreaded sequelae in brain trauma and may present as mass lesions or with diffuse swelling of the brain parenchyma. The edema occurs due to blood brain barrier disruption, vasogenic edema occurs due to poor drainage, and cytotoxic edema occurs due to injury to the parenchyma. Following the Monroe−Kellie doctrine, the progress of edema becomes catastrophic on reaching a point of decompensation. Increase in the intracranial pressure (ICP) leads to a reduction in Cerebral Perfusion pressure (CPP) and eventually brain herniation and ischaemia [2]. This makes the rapid and acute management of elevated ICP and the use of surgical techniques like craniotomy and decompressive craniectomy essential lifesaving interventions. Decompressive craniectomy (DC) is a surgical technique that involves removal of skull bone in order to reduce the ICP by allowing the brain to expand in the presence of a space occupying lesion and/or cerebral oedema [3].

The residual bone defect can be repaired after the cerebral oedema subsides either with use of synthetic graft, allografts from a bone bank, or an autologous graft that may be stored following its removal [4]. The use of synthetic materials for cranial reconstruction like titanium mesh, ceramics, Poly(Methyl Methacrylate) (PMMA), and Hydroxyapatite has been an alternative to cryogenic preservation of bone flaps. With the advent of 3-D printing techniques, these have also been able to demonstrate good cosmetic outcomes [5]. However, synthetic materials are still more expensive and unaffordable in lower- and middle-income and resource-limiting settings. This makes bone preservation more appropriate in such situations [6].

The methods of preservation of autologous flap may vary. Bone grafts are usually stored in a deep freezer at −80 °C, especially in more developed parts of the world. Bone banks with extremely low temperatures have also been shown to be beneficial for the long-term storage of allografts [7]. However, in the context of lower- and middle-income countries (LMICs) and other low resource settings, the use of more conventional freezers with temperature as high as −18 °C are noted [8]. Such high temperatures are further associated with higher risk of surgical site infection, as contamination and growth of flora may not be adequately suppressed [9]. The autoclave and other sterilization techniques before the cranioplasty can further cause the loss of tissue viability [10]. The use of subcutaneous preservation of bone flaps has been noted as far back as the World Wars [11]. Studies have repeatedly shown similar, if not more complications in the use of cryopreservation when compared to subcutaneous preservation [12,13].

Whenever deep cryogenic preservation is not feasible at −80 °C, the use of more conventional freezers is more commonly seen [13]. However, there may be frequent infrastructure failures leading to loss of tissue viability, especially in LMICs like India [14]. The present study aimed to compare the risks, benefits, and outcomes in the use of subcutaneous preservation of bone flaps with cryogenic preservation of bone flaps using a conventional freezer in repair of defects of decompressive craniectomy performed for traumatic brain injury.

The objectives of the paper were to compare the outcomes of subcutaneously preserved bone flaps and cryogenically preserved flaps for autologous cranioplasty.

## 2. Materials and Methods

The study was designed as a single-center, open randomized controlled trial in which patients who underwent decompressive craniectomy for traumatic brain injury were recruited. The study was conducted from July 2023 to December 2024 over a period of 18 month after the approval from the institutional ethics committee on 21 June 2023.

Adult patients (above the age of 18 years) who presented to the Emergency Room of the Department of Trauma Surgery and Critical with traumatic brain injury requiring decompressive craniectomy during the approved period were included in the study after obtaining a written informed consent. Consent was obtained directly from patients who were conscious and alert and from the patient’s guardian or immediate relative in case of altered sensorium or consciousness. Patients who were hemodynamically unstable at the time of presentation, patients who had any loco-regional injury or infection that prevented the use of subcutaneous storage, patients with penetrating or open brain injury, unusable/contaminated autologous flaps, or patients with any other operative contraindications were excluded from enrollment.

Following enrollment, the patient was allocated into group “A” or group “B”. The allocation was done via simple randomization with the help of a sealed paper envelope. Patients who allocated to group “A” had their bone flaps cryopreserved in a conventional freezer at −18 °C (CP group), and those allocated to group “B” had their bone flaps preserved subcutaneously in the anterior abdominal wall (SC group). Based on studies, the complication rate in subcutaneous group was assumed to be 5% as compared to 16% in cryopreserved group [12]. Hence, to declare subcutaneously the preserved group was not inferior to that for the cryopreserved group at a 10% margin of non-inferiority, the minimum sample size for each group was calculated to be 42 (84 in total), in order to achieve a power of 80% and a level of significance of 5%. Anticipating the mortality rate of 26.4% in cases of traumatic brain injury requiring decompressive craniectomy [15], due to patients being lost to follow-up, becoming unfit for further surgical intervention, or bone flap infection before intervention at the storage site, the number of patients enrolled was increased by 46 cases. A minimum of 65 patients were estimated to be enrolled in each group with a total of 130 patients in order to determine any non-inferiority. A total of 158 patients were enrolled by the end of the final enrollment date, and 104 patients were included for the final analysis.

### 2.1. Procedure

Following pre-operative preparation, patients with traumatic brain injury were planned and underwent fronto-temporo-parietal decompressive hemi-craniectomy under general anesthesia. The approach was made using the question mark incision, and the scalp hemostasis was secured using Raney’s clips. A total of four burrs were made at the frontal, parietal, and temporal regions of the skull bone and were connected. The skull bone was carefully removed and handed over for preservation.

For cryopreservation, the bone flaps were removed and washed with hydrogen peroxide and normal saline immediately. Any excess soft tissue or debris was removed. The bone was packed and labeled appropriately with the name, age, gender, and CR number of the patient and stored immediately in a conventional freezer at −18 °C. Subcutaneous preservation was carried out during the same setting as the DC. The patient’s anterior abdominal wall was painted and draped. Incision was made over the skin in the right or left upper or lower quadrants, and the subcutaneous plane was reached. The subcutaneous fat was dissected from the abdominal fascia, and a dead space was made. The bone was debrided, and any sharp corners were nibbled using bone rongeur. The bone and the subcutaneous space were irrigated, and hemostasis was confirmed within the space. Bone was placed, and the skin was closed.

The decompression procedure was completed by opening the dura, evacuating any hemorrhage or foci, and repairing the dura in a lax fashion with the help of grafts harvested from pericranium or temporalis fascia. The patient was transferred to the Neuro-trauma ICU, and post-operative care was provided according to the protocol. Patients were extubated if either sufficient consciousness was gained or elective tracheostomy was carried out in order to wean off mechanical ventilatory support. Patients were discharged after stabilization and advised to follow up for cranioplasty in the trauma OPD after 3 months.

### 2.2. Cranioplasty

**Figure 1:**
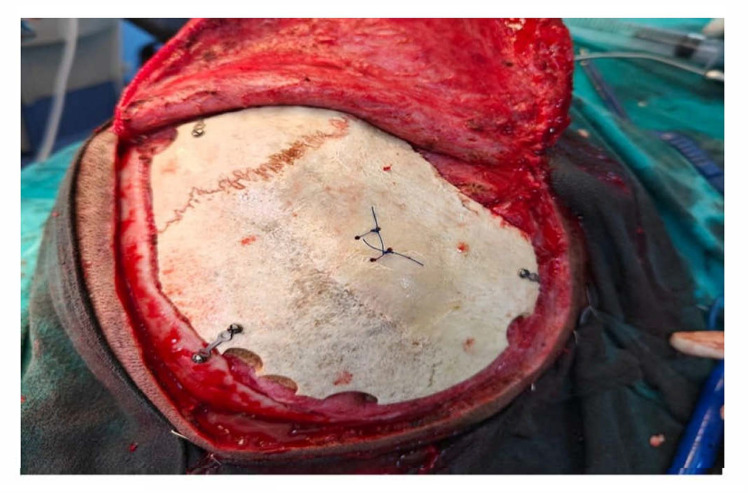
Cranioplasty fixed with titanium miniplates.

Following decompressive craniectomy, the patients were called back telephonically or via outpatient visits after a period of 3 months. The patients was evaluated for surgical fitness and underwent non-contrast computed tomography of the head (NCCT Head), and their GOS scores were noted. The cryopreserved bones were autoclaved one day before cranioplasty while subcutaneously preserved bone flaps were removed at the same time as the intervention. The scalps were opened in layers via the site of the previous incision with careful dissection. Bones were washed thoroughly with normal saline. The bone grafts were placed over the defect and fixed with titanium miniplates (DePuy Synthes, Mumbai, India) (Figure 1). The patients were followed up for one month with regular OPD visits and were assessed for outcomes.

Outcomes were assessed at post-operative day 7 and on post-operative day 30. The outcomes were divided into primary and secondary outcomes. Primary outcomes were assessed as surgical site infection and GOS. The secondary outcomes including the drain output, seroma formation, and reoperation rate were assessed. The final data analysis was performed according to the protocol. Any enrolled patients who expired during the study period, were lost to follow-up, became unfit for further surgical intervention or had bone flap infection at the site of storage before cranioplasty were excluded from the study. Data were tabulated in a spread sheet, and statistical analysis was performed. Means, medians, and standard deviations were calculated for quantitative variables where applicable. In order to determine any correlation between the variables, chi-square tests of significance were performed. A *p*-value less than 0.05 was considered to be significant.

## 3. Results

**Figure 2:**
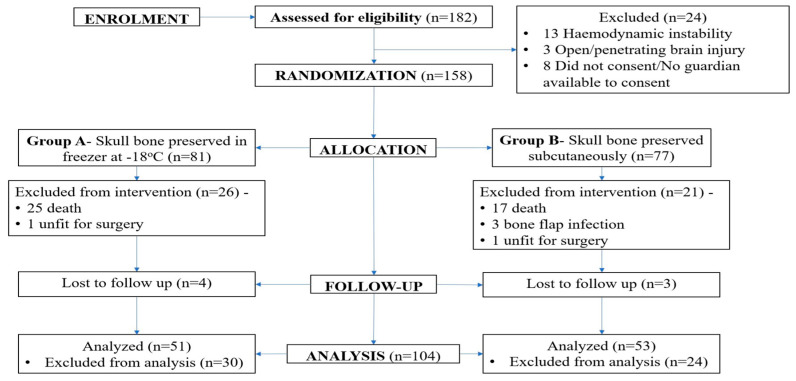
CONSORT diagram of the study.

One hundred and eighty-two adult patients requiring decompressive craniectomy were assessed for eligibility during the study period. A total of 158 patients were enrolled in the study, with 81 patients in group A and 77 patients in group B. Out of the 158 patients, 104 (65.8%) patients were included in the final analysis with 51 patients in group A and 53 patients in group B. Fifty-four patients were excluded from the final analysis, with 42 (26.6%) patients having died during the intervention period, 7 (5.7%) patients lost to follow-up, 2 patients having to discontinue intervention due to poor surgical fitness, and 3 patients excluded due to pre-operative bone flap infection. The consort diagram is shown in Figure 2.

### 3.1. Descriptives

As depicted in Table 1, the minimum age of patients in the study was 18 years, and the maximum age was 74 years with a mean of 35.6 years (SD = 13.8); 67.2% of the population was under the age of 40 years. The gender distribution showed a male preponderance with 73 patients (70.2%) being male. The male-to-female ratio was 2.35:1. The affected side was the left in 47 patients (45.2%) and the right in 57 patients (54.8%). The minimum duration taken from craniectomy to cranioplasty was 89 days while the maximum duration was 105 days, with a median duration of 92 days (IQR: 5.0). Seventeen patients had a pre-operative Glasgow Outcome Scale (GOS) score of 5; 63 patients (60.6%) had a GOS score of 3 or 4, and 12 patients had a GOS score of 2.

The minimum drain output in the study was 10 mL while the maximum was 105 mL, with the mean of 42.3 mL (24.4). Twenty-nine patients of group A and four patients of group B had either seroma and/or surgical site infection, with 31.7% having some form of complication. Sixteen patients from group A and three patients from group B had surgical site infection. Eighteen patients from group A and four patients from group B were found to have seroma. One patient from each of groups A and B underwent repeat surgery. In both cases, it was due to skin flap necrosis and development of epidural abscess. Out of 104 patients, 17 had a pre-operative GOS score of 5 and hence could not be assessed for any further improvement. Out of the 87 remaining patients, 55 (63.2%) had an improvement of GOS score of at least 1 point over a period of 1 month while 36.8% did not show any improvement over that period.

### 3.2. Analysis

The chi-square tests of association were conducted (Table 2) in order to evaluate the outcomes between both the groups. In cases of surgical site infection, the test yielded a (*χ*2) value of 11.5 with 1 degree of freedom and a *p*-value of less than 0.001, and the relative risk of surgical site infection in cryopreserved flap was found to be 5.54 times higher compared to the subcutaneously preserved bone flap. The same test was also carried out in the 87 patients whose GOS scores could be assessed. The value came to be 2.0 with a degree of freedom of 1 and a *p*-value of 0.157, which showed no significance. Chi-square tests of association were also conducted to evaluate the secondary outcomes. Differences in seroma formation were found to be significant with a *p*-value less than 0.001. The association between the method of storage and re-operation rates was not found to be significant (*p* = 0.978). The differences in overall complications were found to be statistically significant (*p* < 0.001). After conducting a chi-square test in order to determine any association between SSI and seroma formation, the *p*-value was found to be 0.013, showing significance.

## 4. Discussion

Cranioplasty is an important step in surgical management of cases of traumatic brain injury who have undergone decompressive craniectomy. It is associated with greater outcomes with regards to the Glasgow Outcome Scale and cosmesis [16]. Cryopreservation at far lower temperatures has been the preferred practice in the context of higher-income countries with a readily available bone bank and advanced health infrastructure. However, the studies that have been conducted generally show that the use of subcutaneously preserved bone flaps tends to have complication rates that are either equivocal or lesser [12,13,17,18].

A total of 158 patients were enrolled in the present study, out of which only 104 were included in the final statistical analysis. Forty-two patients were excluded due to the mortality during the study period, with an observed mortality rate of 26.6% in cases of patients undergoing decompressive craniectomy. This is similar to the study by Huang Y. H. et al. (2013), where a mortality of 26.4% was observed among cases of decompressive craniectomy [15]. Studies like the DECRA trial have demonstrated a slightly lower mortality rate following decompressive craniectomy with a 6-month mortality of 19% [19]. The variation in mortality may be attributable to immature trauma systems and poor health infrastructure in the current study as compared to the West [14].

The mean age of participants in our study was 35.6 ± 13 years with 70.2% male and 29.8% female. The age and gender distribution were similar to the demographic findings by Shafiei M. et al. (2021), where the mean age was 35.53 ± 15.22 years [13]. Studies from more developed and higher-income countries report a larger mean age of their cohorts with Cheng et al. reporting a mean age of 47.55 ± 20.25 years [17]. Younger males tend to be more prone to injury, due to risk-prone behaviors and higher risk during transit or occupation. The studies conducted in more developed areas as shown by Inamasu J et al., Cheng C. H., et al., Al Salihi M. M. et al., and Rosinski et al. showed a higher median age. However, male preponderance was common among all the studies [12,17,20,21].

Most studies from the literature review used cryopreservation at a temperature far lower than −18 °C, from −40 °C to −70 °C [17,21]. The high variability in the temperature used for storage significantly affects the outcome. Shafiei M. et al. (2021) and Robles L.A. et al. (2024) preserved the bones at a higher temperature at −18 °C and used a conventional refrigerator in order to achieve this [8,13]. Other methods of preserving the bone flap by autoclaving or using ethylene oxide and storing them at room temperature have also been documented [21]. In the absence of a deep freezer, conventional freezers and even storage at ambient temperature can be preferable, especially in LMICs and in more isolated settings [8].

The duration between craniectomy and cranioplasty is associated with complication rates. The time duration taken from craniectomy to cranioplasty fell between 89 and 105 days with a median flap storage duration of 93.5 days (3.07 months). In other studies, the mean flap storage duration was reported as 1.25 months (SD = 0.7) by Inamasu, et al., 2.04 months (SD = 1.6) by Cheng, et al., and 5.16 months (SD = 1.87) by Shafiei, et al. [12,13,17]. A longer duration of bone storage has generally been associated with poorer tissue viability and increased risk of infection [7,9,10].

The site of storage in our study was chosen to be the anterior abdominal wall. The option of storing the bone flap in the lateral thigh was also kept in the event of any loco-regional contraindications; however, the site of storage remained consistent in all the patients recruited. Inamasu et al., Rosinski, et al., and Shaifei, et al. also conducted their studies with the site of storage chosen to be the anterior abdominal wall [12,13,21]. In the case of Cheng et al., the site of storage was chosen to be the lateral thigh [17]. There is no evidence to suggest that the specific site of storage drastically changes or affects the outcomes in patients of cranioplasty.

Robles L. A. and Morell (2024) demonstrated that use of conventional freezers at −18 °C was an alternative to the energy-demanding special freezers with similar incidences of aseptic bone resorption and infection [8]. Bhaskar I. P. et al. (2011) showed that bones that are frozen conventionally tend to lose their osteoblastic activities over longer periods of time but can retain the same over shorter intervals [9]. Studies have been conducted on the biomechanical properties of human skull bone and the effects of freezing and thawing them. In one of them, postmortem bone samplings were performed after an interval of 7 days involving 105 cadaver skulls. Biomechanical stress using a three-point bending apparatus was carried out. The study showed that regardless of the time interval of freezing, the biomechanical properties of the skull remain the same after thaw [22].

Up to 20% of cryopreserved skull bones may be microbially contaminated. The greater sensitivity in detecting microbial growth in bone biopsy specimens has been demonstrated in a comparison between superficial swabs and bone biopsy culture [9]. Some investigations have shown positive cultures of up to 50% even when superficial swabs show growth, although the specificity is reduced because of cross-contamination by skin flora such P. acnes and coagulase-negative staphylococci. Because of this, bone biopsies are a better way to find microbial contamination [23,24]. The type of freezing (conventional vs. deep freeze), storage time, and disinfection techniques all affect the rate of bacterial contamination and tissue viability. Bones autoclaved following extended deep freezing have been shown to have a surgical site infection rate of up to 38.5% [10].

There are many factors that affect surgical site infection rates, and older patients with poorer glycaemic control are more likely to have greater rates of SSI than younger patients with adequate glycaemic control [13]. The size of the defect, duration to cranioplasty, post-operative wound dehiscence, and post-operative fluid collection are other factors that contribute to infections. It has been discovered that the presence of wound dehiscence and subgaleal collection are more statistically significant predictors of SSI [25,26].

The rate of surgical site infections, combining superficial and deep infections, ranges from 4% to 28.6% [12,13]. In the CP method, SSI rates can range from 4% to 28.6%, while in the SC method, they can range from 0% to 18.2% [12,13,17,21]. In our study, we reported only two cases of deeper bone flap infection and epidural abscess, both of which required bone removal. Shafiei et al. reported skin necrosis and bone flap infections in at least 4% patients each with more cases reported in cryopreserved flap (*p*-value = 0.307) [13]. Singh et al. reported a bone flap infection rate of 2.8% [27]. Our study demonstrated a significantly higher rate of superficial surgical site infections; however, deeper bone flap infections or epidural abscesses were reported less frequently. However, the post-operative follow-up period in our study was only 1 month; hence, the prevalence of infections needs further study.

Most cases of superficial surgical site infections were managed conservatively with intravenous and oral antibiotics. For the cases of post-operative epidural abscess that were noted in our study, emergency bone removal and wash was performed. Both the patients presented on post-operative day 14 and day 20 with complaints of purulent discharge from surgical site and skin flap necrosis, with non-contrast CT of the head showing the presence of an epidural collection. The patients were re-started on intravenous antibiotics and taken for emergency craniotomy. On removal of the bone, extradural purulent and toxic material was noted. Cultures were taken, and treatments were continued appropriately. The pre-operative and intraoperative images of one of the patients are given in Figure S1. Both the patients were discharged and underwent titanium mesh cranioplasty after 3 months. They tolerated the secondary cranioplasty well and remained stable in further follow-ups.

Shafiei et al. (2021) and Inamasu et al. (2010) showed a statistically significant correlation between the use of cryogenic storage as a preservation strategy and higher rates of surgical site infection [12,13]. The opposite trend of higher surgical site infection and complication rates in subcutaneously maintained flaps is demonstrated by studies by Cheng et al. (2014) and Rosinski et al. (2019) [17,21]. However, the temperature for cryopreservation was significantly lower in these investigations, and the discrepancies were not statistically significant [12,13,17,21]. A detailed comparison of various studies comparing the surgical site infection as an outcome in cryopreserved and subcutaneously preserved bone flaps is given in Table S1.

Subgaleal effusion or seroma following cranioplasty is usually observed when the material used is synthetic like Polyetheretherketone (PEEK) or titanium. Rates of subgaleal effusion as high as 85% have been documented [28]. A study investigating infections in a single material like Porus Hydroxyapatite (PHA) with a long-term follow-up, showed an infection rate of 6%, which was significantly lower than cases of autologous bone flaps [29]. However, a systemic review by Cerveau et al. (2023) demonstrated that the infection-related graft failure following cranioplasty was found to be similar in autologous and allogenic grafts [30]. While other factors like bone flap resorption due to poor tissue viability may influence the use of one material over the other, autologous bone is still the most economical, biocompatible, and cosmetically appealing option [29,30].

Subgaleal fluid collection following cranioplasty is usually a minor complication but an important predictor of surgical site infection [31]. A systematic review of complications and risks associated with the use of autologous bone grafts showed a subgaleal seroma/haematoma rate of 5.8% [32]. In our study, seroma was observed in 22 cases (21.1%) with 18 cases where cryopreserved bones were used. The poor survival of the bone flap and its viability following a longer preservation time and use of autoclave may have contributed to the increased reactionary fluid. Our study also demonstrated a significant correlation between the presence of seroma and surgical site infection. The use of subgaleal drains have been shown to reduce the risk of infection following cranioplasty. A logistic regression analysis by Spake C.S. L. et al. (2024) performed retrospectively over 126 patients of autologous cranioplasty showed a reduced infection risk [33]. The negative suction created by the drain has also been shown to help in adapting the scalp flap over the bone or implant and improve cosmesis [34].

When comparing the two methods of bone preservation and GOS improvement at one month, it was discovered that 31 (35.6%) of the patients in the cryopreserved group and 24 (27.5%) of the patients in the subcutaneously preserved group showed improvement. The statistical significance of the differences was not established. Regardless of the implant type, Posti et al. (2018) also showed a gradual improvement in GOS [35]. Nevertheless, their research also showed a higher rate of complications (42% vs. 7%) linked to the neurological outcome (*p* = 0.003), which our study did not find [35]. This improvement in GOS can be explained by the changes in CSF dynamics due to the presence of a calvarial flap. The study by Chun H.J., et al. (2011) has even demonstrated an improvement in GOS with early cranioplasty performed as soon as the cerebral oedema permitted [36]. Another study by Safi S et al. also showed a statistically significant improvement in GOS, especially when performed in cases of post-traumatic craniectomy and performed at an earlier period less than 12 weeks [16].

On reviewing the overall complication rates among the patients undergoing cranioplasty, it was found that complications can be as low as 17.3% and be as high as 36.6% [17,37]. The variability can be accounted for due to the types of complications studied, variations in the surgical techniques, and differences of patient demographics. The overall complication rate in our study was 31.7%. Certain other complications such as epidural abscess and surgical site infections may also occur over long periods of time. Due to the shorter follow-up duration in our study, complications such as bone flap resorption and post-traumatic hydrocephalus could not be adequately seen. The evaluation of patient outcomes using the Glasgow Outcome Scale can also be performed at 3 and 6 months, which was not done in our study due to the shorter follow-up period allotted.

The most common complication that required the repeat surgery in the study for Shafei, et al. was bone flap resorption, followed by post-operative hydrocephalus. Rosinski et al. also reported cranioplasty removal in eight (6.4%) cases [13,21]. Bone flap resorption is a phenomenon associated with bone grafts of poor viability and contributes to the hesitancy in the use of autologous bone grafts. Factors such as age, fragmentation, simultaneous use of shunts, and poor nutrition have been implicated in the cause, but the pathology is still not fully understood [38]. In order to assess post-operative flap integrity, scoring systems like the Flap Integrity Score and the Oulu Score have been developed [38,39]. Our study did not have an insufficiently long follow-up period to successfully assess this as a complication; however, it is important to be kept in mind during the use of autologous grafts.

Post-traumatic hydrocephalus is another complication associated with high morbidity and mortality rates and is more common in severe traumatic brain injury. Patients may present with clinical features such as headache, altered sensorium, seizures, and flap bulge [40]. Diagnosis is usually confirmed using radiological studies like CT scan, MRI, and cerebral perfusion studies. The use of the CSF infusion test and the tap test may help differentiate ventriculomegaly brain bulge from hydrocephalus in the absence of radiological studies. While management using pharmacological suppression of the CSF has been documented, the use of definitive shunts like ventriculoperitoneal shunt before or during cranioplasty can help manage the complication. The development of hydrocephalus following cranioplasty may be managed by more temporary measures [41].

### Limitations

A significant limitation of this study was its shorter follow-up period, which made it challenging to assess long-term problems such post-operative hydrocephalus and bone flap resorption. The study’s exclusion of patients who underwent delayed cranioplasty after the study’s period or who were lost to follow-up may have potentially created selection bias. The use of simple randomization, instead of block randomization, meant that there was no control of variables according to demographic, clinical, and operative findings, which may have contributed to variations in outcomes and further bias. These limitations make it challenging to perform a comprehensive evaluation of the lifetime of the outcomes and the long-term efficacy of the preservation methods.

## 5. Conclusions

For patients who have traumatic brain injury (TBI) and require decompressive craniectomy, cranioplasty is an essential surgical procedure since it greatly enhances both the neurological and aesthetic outcomes. When the cryogenic storage at −18 °C and subcutaneous storage of autologous bone flaps were compared, it was discovered that, despite both successfully restored calvarial integrity and enhanced Glasgow Outcome Scale (GOS) scores, the subcutaneous technique had a lower risk of side effects like surgical site infections (SSIs). Despite its effectiveness, cryopreservation may not be practical in LMICs because of the lack of energy infrastructure. With less expense and fewer difficulties, subcutaneous preservation is a good substitute, especially for healthcare systems with limited funding. There is, hence, a need for context-specific preservation. Further research with longer follow-up periods is necessary to evaluate late-onset complications and refine cranioplasty protocols.

## Figures and Tables

**Table 1:** Descriptive statistics of patients included in the final analysis.

Variable	Cryopreserved Flap (n = 51)	Subcutaneously Preserved Flap (n = 53)	Overall (n = 104)
Mean age in years (SD)	34.4 (12.8)	36.8 (14.8)	35.6 (13.8)
Gender (male:female)	36:15	37:16	73:31
Laterality (left:right)	24:27	23:30	47:57
Median interval from craniectomy to cranioplasty in days (IQR)	93 (4.5)	92 (4.0)	92 (5.0)
Mean length of cranial defect in cm (SD)	14.1 (0.878)	14.1 (0.978)	14.1 (0.925)
Mean breadth of cranial defect in cm (SD)	11.2 (1.13)	11.3 (0.984)	11.2 (1.05)

**Table 2:** Analysis of outcomes among both the groups.

Outcomes (N)		Frequency—N (%)		*p*-Value (RR)
		CP ^+^	SC *	
Seroma (104)	Present	18 (17.3%)	4 (3.8%)	<0.001 (4.68)
	Absent	33 (31.7%)	49 (47.1%)	
Surgical site infection (104)	Present	16 (15.3%)	3 (2.8%)	<0.001 (5.54)
	Absent	35 (33.6%)	50 (48.0%)	
Drain output on post-operative day 1 (104)	Less than 50 mL	33 (31.7%)	40 (38.4%)	0.23
	More than 50 mL	18 (17.3%)	13 (12.5%)	
Requirement for repeat surgery (104)	Absent	50 (48.2%)	52 (50%)	0.978
	Present	1 (0.9%)	1 (0.9%)	
Improvement in GOS (87)	Present	31 (35.6%)	24 (27.5%)	0.157
	Absent	13 (14.9%)	19 (21.8%)	

+, cryopreserved; *, subcutaneously preserved.

## Data Availability

Data have been anonymized and made available in a public repository as shown in the link: https://doi.org/10.7303/syn66227822.

## Supplementary Materials

The following supporting information can be downloaded at: https://www.mdpi.com/article/10.3390/brainsci15050514/s1, Figure S1: (a) Deep space bone infection leading to skin necrosis and (b) Intraoperative image of epidural abscess; Table S1: Comparing the studies according to the type, storage temperature, incidence of Surgical Site infection and *p*-value.

## Abbreviations

The following abbreviations are used in this manuscript:

DC	decompressive craniectomy
GOS	Glasgow Outcome Scale
ICP	intracranial pressure
TBI	traumatic brain injury
LMIC	lower- and middle-income country
NCCT	non-contrast computed tomography
SSI	surgical site infection
PEEK	Polyetheretherketone
