# Supplementary material for: A Randomized Controlled Trial Comparing Subcutaneous Preservation of Bone Flaps with Cryogenic Preservation of Bone Flaps for Cranioplasty in Cases of Traumatic Brain Injury
Source: Brain Sci. 2025 May 17;15(5):514. doi: 10.3390/brainsci15050514 (PMC12110232; doi:10.3390/brainsci15050514)
Supplement: Supplementary file 1 [file brainsci-15-00514-s001.zip › brainsci-3615363-supplementary.pdf]

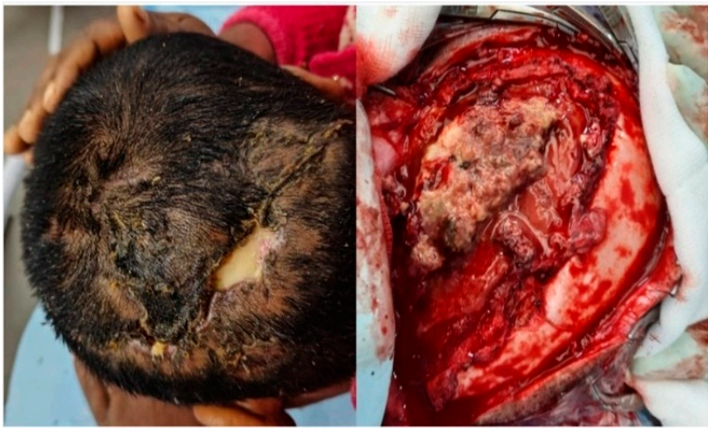

Figure S1: (a) Deep space bone infection leading to skin necrosis and; (b) Intraoperative image of epidural abscess.

Table S1: Comparing the studies according to the type, storage temperature, incidence of Surgical Site infection and p-value

Study	Study type	Storage Temperature (Celsius)	SSI[a]		p value
			CP[b]	SC[c]	
**Current Study**	**Randomized Controlled Trial**	**-18°C**	**16 (15.3%)**	**3 (2.8%)**	**<0.001**
Inamasu, et al., [12]	Retrospective Cohort Study	-70°C	20 (28.6%)	0 (0%)	**0.02**
Shafiei, et al., [13]	Randomized Controlled Trial	-18°C	4 (4%)	0 (0%)	**0.041**
Cheng, et al., [17]	Retrospective Cohort study	-70°C	20 (11.1%)	20 (18.2%)	0.129
Rosinski, et al., [21]	Retrospective Cohort Study	-40°C	6 (10.2%)	7 (20.0%)	0.14

[a] Surgical Site Infection; [b] Cryopreserved; [c] Subcutaneously preserved
